# Thickness Dependence of Ferroelectric and Optical Properties in Pb(Zr_0.53_Ti_0.47_)O_3_ Thin Films

**DOI:** 10.3390/s19194073

**Published:** 2019-09-20

**Authors:** Jian He, Fen Li, Xi Chen, Shuo Qian, Wenping Geng, Kaixi Bi, Jiliang Mu, Xiaojuan Hou, Xiujian Chou

**Affiliations:** 1 Science and Technology on Electronic Test and Measurement Laboratory, North University of China, Taiyuan 030051, China; drhejian@nuc.edu.cn (J.H.); S1706037@st.nuc.edu.cn (F.L.); S1706010@st.nuc.edu.cn (X.C.); qianshuo@nuc.edu.cn (S.Q.); wenpinggeng@nuc.edu.cn (W.G.); bikaixi@nuc.edu.cn (K.B.); mujiliang@nuc.edu.cn (J.M.); houxiaojuan@nuc.edu.cn (X.H.); 2 Taiyuan Heavy Machinery Group Co., Ltd., Taiyuan 030024, China

**Keywords:** PZT, thickness effect, optical properties, ferroelectric properties

## Abstract

As a promising functional material, ferroelectric Pb(Zr*_x_*Ti_1−*x*_)O_3_ (PZT) are widely used in many optical and electronic devices. Remarkably, as the film thickness decreases, the materials’ properties deviate gradually from those of solid materials. In this work, multilayered PZT thin films with different thicknesses are fabricated by Sol-Gel technique. The thickness effect on its microstructure, ferroelectric, and optical properties has been studied. It is found that the surface quality and the crystalline structure vary with the film thickness. Moreover, the increasing film thickness results in a significant increase in remnant polarization, due to the interfacial layer effect. Meanwhile, the dielectric loss and tunability are strongly dependent on thickness. In terms of optical properties, the refractive index of PZT films increase with the increasing thickness, and the photorefractive effect are also influenced by the thickness, which could all be related to the film density and photovoltaic effect. Besides, the band gap decreases as the film thickness increases. This work is significant for the application of PZT thin film in optical and optoelectronic devices.

## 1. Introduction

Ferroelectric Pb(Zr*_x_*,Ti_1−*x*_)O_3_ (PZT) thin films are extremely attractive for the ferroelectric random access memory, optical modulator, photoelectric switch, optical waveguide structure, and UV detection owing to the excellent optoelectronic and electrical properties [1,2,3,4,5,6]. Ranging in thickness from tens of nanometers to tens of microns, high quality PZT films are needed for a variety of applications [7]. Thus, the film thickness is a crucial factor affecting device performance besides some other factors including Zr/Ti ratio [8], annealing temperature [9], preparation method, and other factors [8,9,10,11,12,13,14]. Moreover, the thickness effect would become more pronounced when the films’ thickness vary from micrometer to nanometer scale [15]. Previous investigations mainly focus on the study of PZT films’ thickness effect on microstructure and ferroelectric properties [16,17,18,19]. Ong et al. found that tensile stresses decrease and the dielectric constant increases with the increasing thickness of the PZT film, ranging from ~100 to ~500 nm [16]. Perez et al. found that as the film thickness increases from ~140 to ~700 nm, the coercive electric field decreases, while the grain size and the piezoelectric coefficient (*d*_33_) increase [17]. However, as a promising material in optoelectronics field, it is necessary to study the thickness dependence of the optical properties of ferroelectric PZT thin film. Studying Pb(Zr_0.2_Ti_0.8_)_0.70_Ni_0.30_O_3−*δ*_ (PZTNi30) thin films in the thickness range of 5–400 nm, Shalini and co-workers found the bandgap can change regularly with thickness, and considered the PZTNi30 as a ferroelectric photovoltaic material [20]. Lappalainen et al. also found the bandgap increased as the thickness of Pb_0.97_Nd_0.02_(Zr_0.55_Ti_0.45_)O_3_ film decreased from 465 to 80 nm, with regular changes in refractive index and absorption coefficient [21].

In this work, Pb(Zr_0.53_Ti_0.47_)O_3_ thin films with different thickness were prepared using Sol-Gel technique. We characterized and analyzed systematically its surface morphology, crystal phase, hysteresis loops, permittivity, and optical constants. The thickness effect on the ferroelectric and optical properties of PZT thin film is examined. Results shows that film thickness is an important factor affecting the structure and properties of film.

## 2. Experimental Sections

### 2.1. Experimental Details

**Figure 1:**
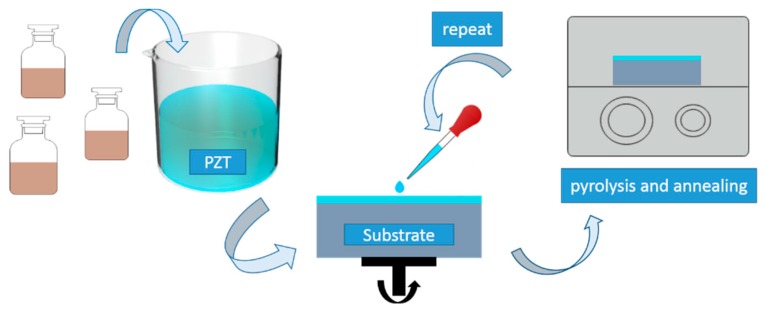
The schematic diagram of sol-gel process of Pb(Zr_0.53_Ti_0.47_)O_3_ film.

Pb(Zr_0.53_Ti_0.47_)O_3_ (PZT) thin film were prepared using the Sol-Gel technique with spin-coat process. Firstly, the 0.4 M precursor solution was obtained by mixing lead acetate trihydrate, zirconium propoxide, titanium isopropoxide, and some organic solvent including lactic acid, acetic acid, glycol ether, and ethylene glycol in a certain proportion, with a Zr/Ti ratio of 53/47. Then, the precursor was dripped onto the substrate and spin-coated at 3000 rpm for 30 s. Finally, pyrolysis and annealing processes were carried out at 350 °C for 10 min and 650 °C for 30 min respectively, using Tube annealing furnace (BTF-1200C, AnHui BEQ Equipment Technology CO., Ltd., Hefei, China) in atmospheric conditions to obtain PZT films with crystalline phase [22,23]. The schematic diagram of film preparation is shown in Figure 1. Control the number of spin-coating to get a desired thickness, and each coating is about 100 nm thick. For simplicity, the film samples are labeled as PZT-*n* (*n* is coating number, which is equal to 1, 2, 3, 4, 6, 8). The film sample PZT-1, PZT-2, and PZT-3 were deposited on mica substrates for ellipsometry measurements, and PZT-4, PZT-6, and PZT-8 were prepared on Pt/Ti/SiO_2_/Si substrates with Au top electrode (diameter of 500 μm), fabricated by magnetron sputtering for electrical measurements. It is because the mica absorbs light weakly and more layers will cause difficulties in elliptical fitting but good for getting a convinced hysteresis-loop.

### 2.2. Experimental Methods

The crystalline structure of PZT thin films was detected by an X-ray diffractometer (DX-2700) with Cu Kα radiation. The surface morphology was characterized by piezoresponse force microscopy (Asylum Research, MFP-3D, Goleta, CA, USA) and scanning electron microscope (FE-SEM, SUPPA-55, Zeiss, Germany). Ferroelectric response, including hysteresis loops and permittivity of PZT films were obtained by a ferroelectric test system (TF Analyzer 2000E, axiACCT Systems, Aachen, Germany) at room temperature. The optical properties were characterized by a high precision automatic photometric ellipsometer (ME-L, Wuhan Eoptics Technology Co., Wuhan, China). The measurements were carried out at room temperature, using speckles with diameter of 200 μm, operated at an angle of incidence of 60° and wavelength range of 193–1690 nm. Dielectric constant and optical constant were obtained by data fitting using Tauc-Lorentz dispersion model.

## 3. Results and Discussion

### 3.1. Microstructure and Morphology

**Figure 2:**
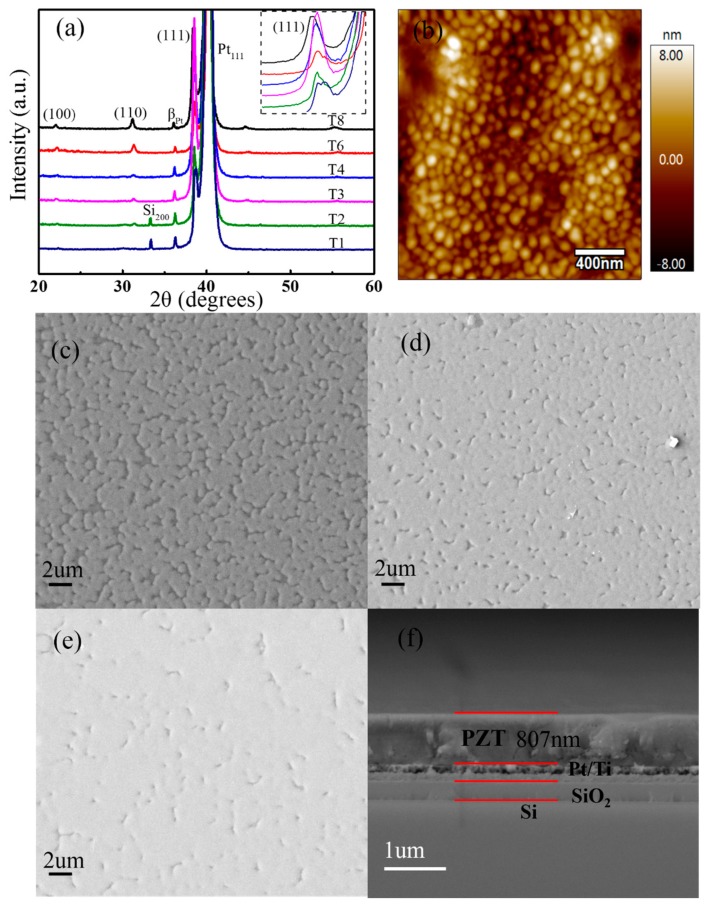
(**a**) XRD pattern of PZT films prepared on Pt/Ti/SiO_2_/Si substrates with different thicknesses *a*. (**b**) AFM surface morphology in area of 2 μm × 2 μm of PZT-8. Top-view of (**c**) PZT-4, (**d**) PZT-6 and (**e**) PZT-8 and (**f**) cross-sectional SEM images of PZT-8. The inset of Figure 2a presents the local enlargement of (111) orientation.

The X-ray diffraction pattern shown in Figure 2a reveals that all the PZT film deposited on Pt/Ti/SiO_2_/Si substrates have good perovskite structures, and present different orientations, among which (111) are preferentially oriented [24,25,26]. It can be seen from the inset of Figure 2a that the intensity of preferred (111) diffraction peak has no obvious trend with the increasing thickness. The exact reason needs further investigation in future. The PZT films with a Zr/Ti ratio of 53:47 is near the morphotropic phase boundary, and can have a preferred orientation (111). All the root mean square (RMS) roughness values of the PZT thin films with thickness from ~100 to ~800 nm are about 3 nm, and AFM surface morphology (scanning area is 2 μm × 2 μm) of PZT film at ~800 nm is presented in Figure 2b. Figure 2c–e gives the top-view images of PZT-4, PZT-6, and PZT-8. It can be seen that the film surface is smooth without cracks, and becomes denser as the coating layers increase. Figure 2f shows the clear interfacial structure and the thickness (about 800 nm) of PZT-8. That conforms to the experience of ~100 nm thick per coating layer.

### 3.2. Ferroelectric Responses

**Figure 3:**
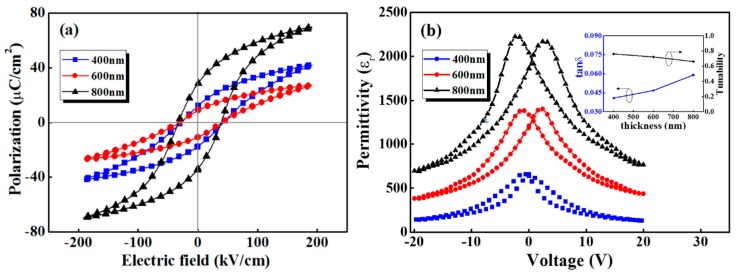
(**a**) Hysteresis loops and (**b**) permittivity *vs* voltage curves of PZT film with different thicknesses. The inset of (**b**) present dielectric loss and dielectric tunability of PZT film with different thickness.

Figure 3a shows hysteresis loop of the PZT films with different thicknesses at 100 Hz, demonstrating a good ferroelectric nature. There is remnant polarization shift to different extent, which originates from the electrode-induced different contact barrier between the upper and lower electrode-film interface [27]. It can be seen that remnant polarization (*P_r_*) increases drastically with the increasing film thickness, while the coercive electric field keeps constant. Especially as the film thickness varies from 400 to 600 nm and then to 800 nm, the remnant polarization increases by 51.61% and 106.98%, respectively. Generally, leakage current, interface layer, and substrate clamping effect are several factors to be considered [15,17,28]. With the increasing film thickness, the influence of leakage current on remnant polarization decreases. In addition, the interface layer and substrate clamping effect would be weakened, resulting in the increasing ferroelectric polarization. 

Figure 3b presents the permittivity (*ε_r_*) curves of PZT-4, PZT-6, and PZT-8, which is obtained based on the capacitance measurement using ferroelectric test system and the equation $\varepsilon_{r}=\frac{C\cdot d}{\varepsilon_{0}\cdot s}$. In the equation, the *C* and the *d* represent the capacitance and film thickness, respectively. The $\varepsilon_{0}$ and the *s* are vacuum permittivity and electrode area, respectively. Obviously, the permittivity curve shows a typical butterfly shape with two maxima related to the coercive voltage. Additionally, the permittivity has a great relationship with film thickness. The maximum dielectric tunability and FOM value are calculated according to the following formulas [26]

(1) $$\mathrm{Tunability}=\frac{\varepsilon_{n}-\varepsilon_{E}}{\varepsilon_{n}}$$

(2) FOM=Tunability/tanδ

where tanδ is the dielectric loss, $\varepsilon_{n}$ and $\varepsilon_{E}$ represent the permittivity without and with the applied electric field *E*, respectively. The inset in Figure 3b shows that dielectric loss tanδ increases with the increasing film thickness, while the dielectric tunability decreases. So, it is inferred that FOM value would decrease. Meanwhile, the permittivity, dielectric loss, and dielectric tunability of PZT film are strongly dependent on the film thickness. This phenomenon can be attributed to the low dielectric constant layer between metal electrode and PZT thin film. That means the interface would lead to a remarkably decreasing permittivity for the PZT film with a lower thickness, while such influence will decrease with increasing thickness [15]. The improved PZT thin films with the increasing density would lead to a lower leakage current, which can also explain the increase of permittivity [29,30,31].

### 3.3. Optical Properties of PZT Film

**Figure 4:**
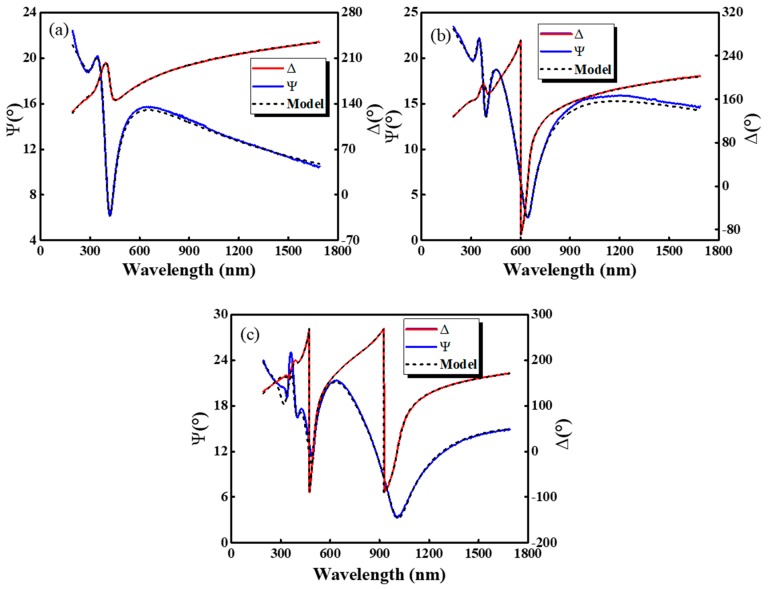
Spectra of the ellipometric parameters (**a**) *Ψ* and (**b**) Δ at room temperature as functions of wavelength for the PZT thin films with different thickness at (**a**) 90 nm, (**b**) 164 nm and (**c**) 252 nm.

The experimental data obtained by ellipsometry are the angles *Ψ* and Δ, which can characterize the structural and optical properties of materials, are defined by

(3) $$\rho =r_{p}/r_{s}=\tan\Psi e^{i\Delta }$$

where $r_{s}$ and $r_{p}$ represent the Fresnel reflection coefficient of the polarized light perpendicular and parallel to the incidence plane, respectively [32,33]. The experimental data are analyzed by a multi-phase model (air/rough interface/PZT film/mica). The mica and the roughness layer are fitted by Cauchy model and effective medium model respectively. The Tauc-Lorentz model was used for PZT films. Figure 4 presents the obtained experimental (dotted lines) and fitted (solid line) data of PZT film with different thicknesses. It can be seen that the calculated result fits the experimental data well based on the proposed multi-phase model. In the higher energy range, the ellipsometry oscillates, which is because of the multiple reflections from internal interface in film. Additionally the thinner the film, the lower the oscillation frequency. The calculated thickness of PZT films with 1–3 coating layers from the Tauc-Lorentz model are 90, 164 and 252 nm, respectively.

**Figure 5:**
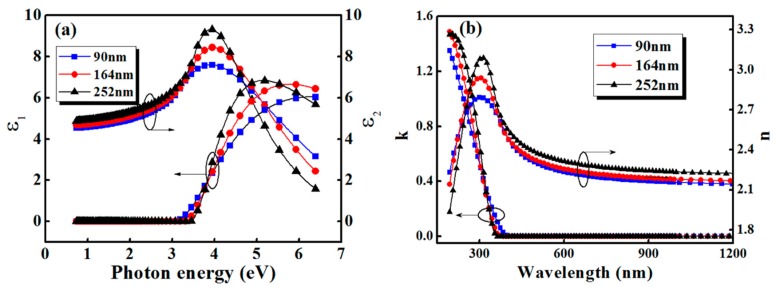
(**a**) Dielectric function and (**b**) (*n*, *k*) spectra of PZT thin film with different thickness calculated from the Tauc-Lorentz model.

Figure 5 shows the optical constant as the function of wavelength in PZT thin film with different thickness. The Tauc-Lorentz model is chosen to model the dielectric function of PZT film. The imaginary part *ε*_2_ is given by the equation [34,35]:

(4) $$\varepsilon_{2}=\frac{A\;En_{0}C{\left(En-E_{g}\right)}^{2}}{{\left(En^{2}-En_{0}{}^{2}\right)}^{2}+C^{2}En^{2}}\frac{1}{En}\;\left(En>E_{g}\right)=0\;\left(En<E_{g}\right)$$

The real part *ε*_1_ is expressed by the following equation:

(5a) $$\begin{array}{cc} \varepsilon_{1}= & \varepsilon_{1}\left(\infty \right)+\frac{AC}{\pi \xi^{4}}\frac{\;a_{ln}}{2\alpha En_{0}}\ln\left(\frac{En_{0}{}^{2}+E_{g}{}^{2}-E_{g}}{En_{0}{}^{2}+E_{g}{}^{2}-E_{g}}\right)-\frac{A}{\pi \xi^{4}}\frac{a_{\tan}}{En_{0}}\left[\pi -tan^{-1}\left(\frac{-2E_{g}+\alpha }{C}\right)\right]\\ & +2\frac{A\;En_{0}}{\pi \xi^{4}\alpha }E_{g}\left(En^{2}-\gamma^{2}\right)\left[\pi +2tan^{-1}\left(2\frac{\gamma^{2}-E_{g}{}^{2}}{\alpha C}\right)\right]\\ & -\frac{A\;En_{0}C}{\pi \xi^{4}}\frac{En^{2}+E_{g}{}^{2}}{En}ln\left(2\frac{\left|En-E_{g}\right|}{En+E_{g}}\right)\\ & +\frac{2A\;En_{0}C}{\pi \xi^{4}}E_{g}ln\left[\frac{\left|En-E_{g}\right|\left(En+E_{g}\right)}{\sqrt{{\left(En^{2}-E_{g}{}^{2}\right)}^{2}+E_{g}{}^{2}C^{2}}}\right] \end{array}$$

where

(5b) $$\;a_{ln}=\left(E_{g}{}^{2}-En_{0}{}^{2}\right)En^{2}+E_{g}{}^{2}C^{2}-En_{0}{}^{2}\left(En_{0}{}^{2}+3E_{g}{}^{2}\right)\;$$

(5c) $${\;a}_{\tan}=\left(En^{2}-En_{0}{}^{2}\right)\left(En_{0}{}^{2}+E_{g}{}^{2}\right)+E_{g}{}^{2}C^{2}$$

(5d) $$\;\xi^{4}={\left(En^{2}-\gamma^{2}\right)}^{2}+\alpha^{2}C^{2}/4$$

(5e) $$\alpha =\sqrt{4En_{0}{}^{2}-C^{2}}$$

(5f) $$\gamma =\sqrt{En_{0}{}^{2}-C^{2}/2}$$

Despite the complexity of the above equations, the dielectric function expression of the model is from those parameters listed in Table 1. The *A* and *C* of the model represent the amplitude and half width of the $\varepsilon_{2}$ peak, respectively. The *ε*_1_(∞) is the dielectric constant when the photon energy is infinite. The *En*_0_ is the constant related to the transition matrix element, and the *E_g_* is the Tauc gap of the Tauc-Lorentz model. The dielectric function is related to both film thickness and photon energy as shown in Figure 5a. The *ε*_1_ increases while the film thickness increases from 90 to 252 nm in the visible-infrared region, which is consistent with the variation trend of the dielectric constant obtained from the permittivity curve. Under UV irradiation, the *ε*_1_ of thicker film decreases much faster than that of a thinner one. It is known that the photoelectric effect of PZT occurs in ultraviolet region, so we can attribute the decay of dielectric constant to the emergence of photon-generated carriers [35].

Figure 5b shows the optical constant curves of PZT film obtained by the relation $N^{2}\equiv \varepsilon$, where $\varepsilon =\varepsilon_{1}-i\varepsilon_{2}$ and N=n−ik. The refractive index *n* in the spectral range of 260–1800 nm increases with the increase of film thickness. At about 305 nm, *n* reach the maximum value of 2.79, 2.94, and 3.07 for the PZT films with thicknesses of 90, 164, and 252 nm, respectively. This phenomenon is generally considered to be related to several factors. On the one hand, according to the growth dynamics of film, the thicker film has a relatively low proportion of voids, and the structure is relatively denser, which makes the refractive index increase with the increasing thickness. What is more, the multi-layered structure of PZT films may have an impact on refractive index, which becomes more obvious as the coating layers increase. In contrast, in the range of wavelengths from 190 to 260 nm, the film refractive index decreases with the increasing thickness. As the incident wavelength becomes shorter, the film refractive index decreases sharply. Meanwhile, the thicker film has a larger change rate. These results could be associated with the photorefractive effect besides the dispersion in PZT film. Photon-generated carriers were produced in the film and diffused under UV irradiation, leading to the establishment of build-in electric field, which induces the electro-optic effect and subsequently the change of refractive index [36,37,38].

**Figure 6:**
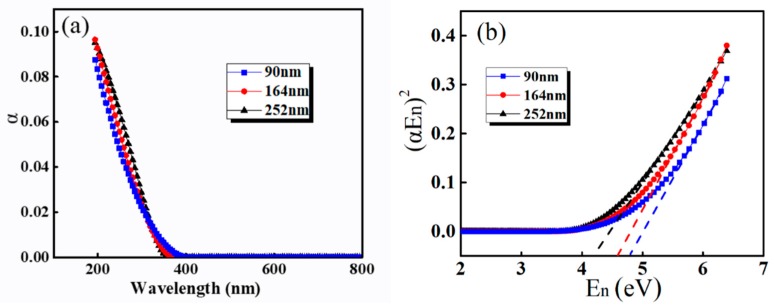
(**a**) absorption coefficient *α* and (**b**) the plot of (*αhv*)^2^ vs. *hv* for PZT film with different thickness.

The absorption coefficient (*α*) and band gap (*E_g_*) are estimated by [39,40,41]:

(6) $$\alpha =\frac{4\pi k}{\lambda }$$

(7) $${\left(\alpha E_{n}\right)}^{m}=K\left(E_{n}-E_{g}\right)$$

where *λ* and *E_n_* represent the wavelength and photon energy of the incident light, respectively, and *K* is a constant independent of the incident light. The m is exponent depending on whether the material is transitioning directly or indirectly. Figure 6a show the *α* of PZT film with different thickness. When the incident wavelength is shorter than 320 nm, the *α* of the thicker film is larger than that of the thinner film. It may be related to the interface layer between the coating layers of PZT films. The plot of (*αE_n_*)^2^ vs *E_n_* is shown in Figure 6b. Because of the direct transition in PZT, the *E*_g_ is obtained by linearly extrapolating of the (*αE_n_*)^2^ plot to zero. The result shows that the *E*_g_ decreases from 4.78 to 4.2 eV, with the film thickness increasing from 90 to 252 nm, which are related to the reduction of defect states, which would increase the probability of transition [42]. 

## 4. Conclusions 

In summary, the thickness of PZT thin film would significantly affect its microstructure, optical, and electrical properties. With the thickness increase, the preferred (111) orientation is stronger to some extent. The hysteresis loops and permittivity are greatly affected by the film thickness, especially the remnant polarization *P_r_,* which increases by 213.8% as the film thickness changes from ~400 to ~800 nm. When the film thickness increases from 90 nm to 252 nm, the refractive index increases in spectral range of 260–1800 nm and decreases in range of 190–260 nm because of the multilayered film density and photoelectric effect. In addition, the dielectric constant and band gap are strongly dependent on film thickness. This work is of great significance for the regulation of electrical and optical properties.

## Figures and Tables

**Table 1:** The values of the analytical parameters from the Tauc-Lorentz model for PZT film with different thickness.

Film Thickness (nm)	*A*	*C*	*ε*_1_(∞)	*En*_0_	*E_g_*
90	366.61	8.24	3.15	3.81	3.05
164	495.46	4.47	2.43	3.30	3.29
252	530.13	2.51	1.58	3.42	3.42
